# Synthetic contrast-enhanced computed tomography generation using a deep convolutional neural network for cardiac substructure delineation in breast cancer radiation therapy: a feasibility study

**DOI:** 10.1186/s13014-022-02051-0

**Published:** 2022-04-22

**Authors:** Jaehee Chun, Jee Suk Chang, Caleb Oh, InKyung Park, Min Seo Choi, Chae-Seon Hong, Hojin Kim, Gowoon Yang, Jin Young Moon, Seung Yeun Chung, Young Joo Suh, Jin Sung Kim

**Affiliations:** 1 Department of Radiation Oncology, Yonsei Cancer Center, Yonsei University College of Medicine, Seoul, South Korea; 2 Medical Physics and Biomedical Engineering Lab (MPBEL), Yonsei University College of Medicine, Seoul, South Korea; 3 Oncosoft Inc, Seoul, South Korea; 4 Department of Radiology, Yonsei University College of Medicine, Seoul, South Korea

**Keywords:** Contrast-enhanced computed tomography, Deep learning, Radiation therapy, Breast cancer, Radiation-induced heart disease

## Abstract

**Background:**

Adjuvant radiation therapy improves the overall survival and loco-regional control in patients with breast cancer. However, radiation-induced heart disease, which occurs after treatment from incidental radiation exposure to the cardiac organ, is an emerging challenge. This study aimed to generate synthetic contrast-enhanced computed tomography (SCECT) from non-contrast CT (NCT) using deep learning (DL) and investigate its role in contouring cardiac substructures. We also aimed to determine its applicability for a retrospective study on the substructure volume-dose relationship for predicting radiation-induced heart disease.

**Methods:**

We prepared NCT-CECT cardiac scan pairs of 59 patients. Of these, 35, 4, and 20 pairs were used for training, validation, and testing, respectively. We adopted conditional generative adversarial network as a framework to generate SCECT. SCECT was validated in the following three stages: (1) The similarity between SCECT and CECT was evaluated; (2) Manual contouring was performed on SCECT and CECT with sufficient intervals and based on this, the geometric similarity of cardiac substructures was measured between them; (3) The treatment plan was quantitatively analyzed based on the contours of SCECT and CECT.

**Results:**

While the mean values (± standard deviation) of the mean absolute error, peak signal-to-noise ratio, and structural similarity index measure between SCECT and CECT were 20.66 ± 5.29, 21.57 ± 1.85, and 0.77 ± 0.06, those were 23.95 ± 6.98, 20.67 ± 2.34, and 0.76 ± 0.07 between NCT and CECT, respectively. The Dice similarity coefficients and mean surface distance between the contours of SCECT and CECT were 0.81 ± 0.06 and 2.44 ± 0.72, respectively. The dosimetry analysis displayed error rates of 0.13 ± 0.27 Gy and 0.71 ± 1.34% for the mean heart dose and V5Gy, respectively.

**Conclusion:**

Our findings displayed the feasibility of SCECT generation from NCT and its potential for cardiac substructure delineation in patients who underwent breast radiation therapy.

**Supplementary Information:**

The online version contains supplementary material available at 10.1186/s13014-022-02051-0.

## Background

Adjuvant radiation therapy (RT) improves the overall survival and loco-regional control in patients with breast cancer [1]. However, radiation-induced heart disease (RIHD), which occurs years after treatment from incidental radiation exposure to the cardiac organ, is an emerging challenge of utmost importance [2]. Darby et al. demonstrated that the risk of an acute coronary event increases linearly with the mean heart dose (MHD) without a safe threshold of exposure [3]. These dose–response relationships were corroborated by subsequent studies on modern 3D data [4].

A recent study suggested that the dose-volume data of cardiac substructure units along with the heart might provide a more accurate prediction of RIHD than MHD [5, 6]. In breast cancer, van den Bogaard et al. identified the volume of the left ventricle receiving 5 Gy (LV-V5) as a more important prognostic dose-volume parameter than MHD for predicting an acute coronary event [6]. In this context, the ongoing MEDIRAD-BRACE study (NCT03211442) is recruiting 7000 participants to validate these findings. Moreover, the ongoing Radiotherapy Comparative Effectiveness randomized clinical trial (NCT02603341) aims to compare the role of proton beam therapy with photon beam therapy in breast cancer and intends to study the dose-volume profiles of cardiac substructures. In contrast, the dose to the left anterior descending (LAD) coronary artery was also suggested as a more reliable surrogate for the risk of major cardiac events than MHD [7].

Considering that cardiac structures are appropriately visualized in an electrocardiography-gated contrast-enhanced CT (CECT) or magnetic resonance imaging (MRI) scan, a bottleneck for more profound and active studies on cardiac structure-dose relationships comprises difficulties in contouring the heart substructure on planning CT scans for breast RT, which are generally obtained without the intravenous (IV) administration of a contrast agent. The use of CECT in the breast cancer staging is not indicated for patients with early breast cancer in the absence of signs/symptoms of metastatic disease according to NCCN guidelines version 2.2022 [8]. The use of breast MRI also is optional and is not universally recommended by experts in the field [8].

To overcome these limitations, some studies have tried to distinguish the heart substructure retrospectively in an environment consisting of non-contrast CT (NCT). Morris et al. developed atlas- and deep learning (DL)-based auto-contouring (AC) pipelines leveraging MRI’s soft tissue contrast, coupled with NCT for cardiac substructure delineation [9, 10]. However, it has a limitation in that additional images must be obtained, which eventually increases the workflow burden of breast RT. More recent studies developed DL models for the AC of the heart and its substructures directly on NCT [11–13]. However, it is mentioned that the manual contours itself, which should be the ground truth of the DL model, may be inaccurate due to the poor visibility of NCT [13]. Moreover, manual modification of prediction from the DL model should be performed on NCT with nothing visible in their studies.

To enable contouring and modification of cardiac substructure without additional image acquisition, it would be advantageous to provide a basis for them rather than the contours themselves. In this study, we aimed to generate synthetic contrast-enhanced CT (SCECT) from NCT using a deep convolutional neural network (DCNN) for cardiac substructure delineation in breast cancer RT.

## Methods and materials

### Study design

**Fig. 1:**
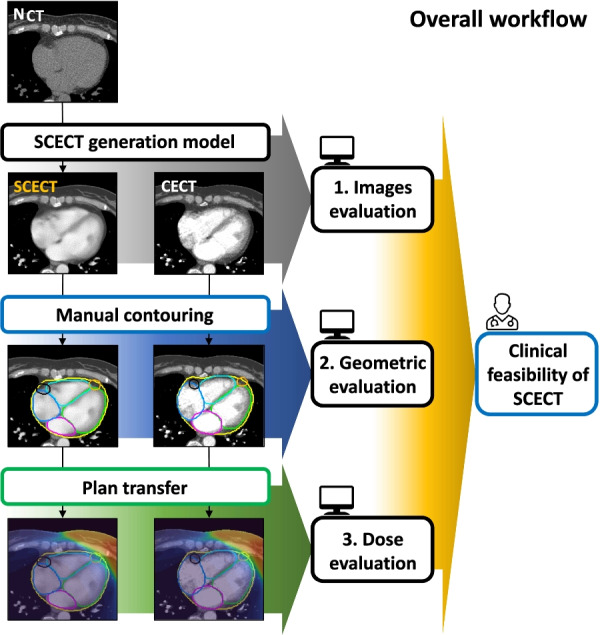
The overall workflow for the validation of synthetic contrast-enhanced computed tomography (SCECT). The validation was conducted in the following three stages: (1) The similarity between SCECT and contrast-enhanced CT (CECT) was evaluated; (2) Manual contouring was performed on SCECT and CECT with interval and based on it, the geometric similarity of cardiac substructures in each image group was measured; (3) The treatment plan was quantitatively analyzed based on the contours of SCECT and CECT

We intended to leverage DL to generate SCECT from NCT and verify its manifold utility. SCECT was validated in four stages, as shown in Fig. 1. (1) The similarity between SCECT generated from the DL model and CECT was evaluated in terms of image quality. (2) The cardiac substructures were manually contoured based on SCECT and CECT with sufficient intervals. Then, the geometric similarity between contours in each image was measured. (3) We quantitatively compared and analyzed the treatment plan based on the contours of SCECT and CECT. This study was approved by the institutional review board of our institution.

### Data preparation

We prepared NCT-CECT cardiac scan pairs of 59 patients comprising an average resolution of ~ 0.8 × 0.8 × 1.0 mm^3^. For all patients, NCT and CECT were scanned by using one of the following multidetector row scanners: Somatom Sensation 16, Somatom Sensation 64, Definition Flash (Siemens Medical Solutions, Forchheim, Germany), Discovery CT 750 HD, Revolution (GE Medical Systems, Milwaukee, Wisconsin, USA), or iCT (Philips Medical Systems, the Netherlands). All cardiac CT scans were acquired in a volumetric mode in full inspiration. A bolus of 50–90 mL (1.5 mL/kg body weight) of iopamidol (300 mg I/mL, Radisense, Taejoon Pharmaceutical, Seoul, South Korea) was injected intravenously at a flow rate of 3 mL/s for CECT images.

### Deep learning-based SCECT generation

The 59 NCT-CECT cardiac scan pairs underwent multiple pre-processing steps for use in the SCECT generation model. First, image resolution was resampled to 0.9 × 0.9 × 1.0 mm^3^. After matching the structure of paired NCT-CECT scans based on the deformable image registration algorithm [14, 15], we cropped the 384 × 84 × 150 area near the heart. Subsequently, the contrast window was consistently set to [− 150, 500] Hounsfield units (HU) for a better analysis of important features to be by the DL model.

**Fig. 2:**
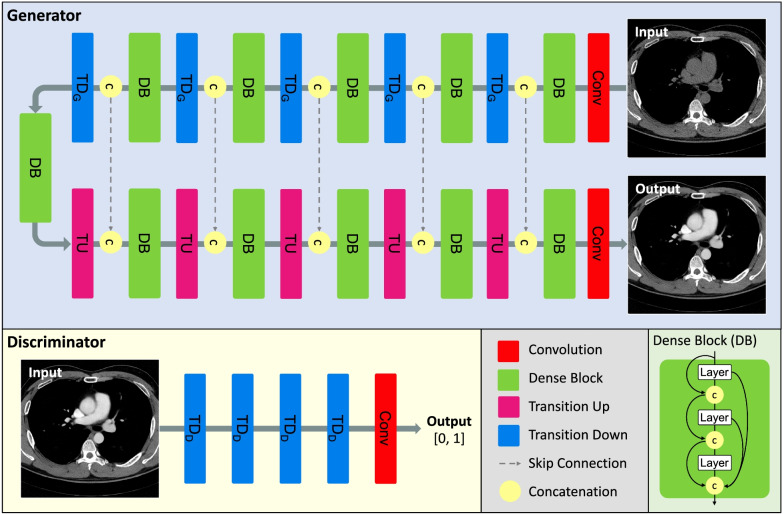
Our architecture diagram for the deep learning-based synthetic contrast-enhanced computed tomography (SCECT) generation model. The generator model has five Transition Down (TD_G_) and Up (TU) structures, and image features are analyzed in depth through Dense Block (DB) at each stage. Information of low and high-level features initially extracted from input image is preserved until the end through skip connection and concatenation. The inpuf of generator is NCT, ground truth is CECT, and predicted output is SCECT. The Discriminator model has four transition down (TD_D_) structures that are slightly different from TD_G_. The input of the discriminator is a two-channel image in which NCT is concatenated with CECT or SCECT, respectively. The ground truth is 0 or 1, and the predicted output is a decimal value between [0, 1]

We used conditional generative adversarial network (cGAN) [16, 17] as our SCECT generation framework. cGAN learns a mapping from observed image $x$ to $y$ $G:x \to y$. The generator $G$ is trained to produce outputs that cannot be distinguished from “real” images by an adversarially trained discriminator, $D$, which is trained to detect the generator’s “fakes.” The objective of a cGAN can be expressed as

1 $${G}^{*}=arg\underset{G}{\mathrm{min}}\underset{D}{\mathrm{max}}\left\{{\mathbb{E}}_{x,y}\mathrm{log}D\left(x,y\right)+{\mathbb{E}}_{x}\mathrm{log}\left(1-D\left(G\left(x\right)\right)\right)\right\} +\lambda {\Vert y-G\left(x\right)\Vert }_{1}$$

where *G* tries to minimize this objective against an adversarial *D* that tries to maximize it. Unlike the original cGAN learned from noise z, in this study, it is learned in an environment similar to almost supervised learning with L1 loss function [17]. *λ* is arbitrary constant variable, and it was set to 100 in this study. The architecture diagram and further details of our models are illustrated in Fig. 2 and Additional file 1: Figure S1. The modified 2D fully convolutional DenseNet (FC-DenseNet) was used as a generator. As a discriminator, that of PatchGAN model was borrowed, and it was modified and used according to the environment of this study.

The input and ground truth image of the prepared 384 × 384 size were randomly cropped to 352 × 352 size and used for training of *G* and *D*, and other data augmentations were not applied. *G* and *D* were learned from scratch. Out of 59 datasets, 35 were used for training, 4 for validation, and 20 for testing. DL model training was conducted by monitoring training and validation datasets, and it was stopped at 200 epochs where the loss values for the validation data were saturated. Additional hyper-parameters for training the SCECT generation model are detailed in Table 1. The 20 testing datasets were independently managed during the training process, and the model application to the testing datasets was executed only once after the model was fully trained.

**Table 1:** Hyper-parameters for training deep learning-based synthetic contrast-enhanced computed tomography generation model

Parameter	Value
No. of parameters	$G$: 5.4 M/$D$: 1.6 M
Batch size	4
Loss function	Adversarial + L1 loss
Optimizers	$G$: Adam/$D$: SGD
Starting learning rate	$G$: 0.0002/$D$: 0.00002
Number of epochs	200

G, generator; D, discriminator; SGD, stochastic gradient descent

We used the mean absolute error (MAE), peak signal-to-noise ratio (PSNR), and structural similarity index measure (SSIM) [18] for evaluating the image quality of the SCECT. The formulas used are as follows:

3 $$MAE = \frac{{\mathop \sum \nolimits_{i = 1}^{n} \left| {y_{i} - x_{i} } \right|}}{n}$$

3 $$PSNR = 10\log_{10} \left( {MAX_{I}^{2} /MSE} \right)$$

4 $$SSIM = \frac{{\left( {2\mu_{x} \mu_{y} + c_{1} } \right)\left( {2\sigma_{xy} + c_{2} } \right)}}{{\left( {\mu_{x}^{2} + \mu_{y}^{2} + c_{1} } \right)\left( {\sigma_{x}^{2} + \sigma_{y}^{2} + c_{2} } \right)}}.$$

Here, $x$ denotes the true value (CECT); $y$, the prediction (SCECT), *MAX*, the maximum possible pixel value of the image; *MSE,* the mean squared error of two images; $\mu$, the average; $\sigma$, the variance or covariance; and $c$, the variable to stabilize the division with weak denominator. The lower the MAE and the higher the PSNR and SSIM, the better the values.

### Manual contouring of cardiac substructures

SCECT generation was aimed at contouring the cardiac structures. Accordingly, manual contouring was performed on SCECT of 20 testing patients by referring to the University of Michigan cardiac atlas [19], and geometric similarity evaluation with the contours of CECT was conducted. The SCECT predicted from the deep learning model is restored to another DICOM file that shares most of the information with the existing DICOM file of the NCT. Image information on areas other than prediction ROI (352 × 352 × 150) shares that of NCT.

MIM Maestro® (MIM Software, Inc.) was used for contouring organs-at-risk. There were seven target substructures for delineation, including the heart, left ventricle, left atrium, right ventricle, right atrium, LAD, and right coronary artery (RCA). A physician with more than 10 years of experience in breast RT delineated the cardiac substructure of testing datasets. In order to prevent possible bias, the contouring of each image group was conducted at intervals of more than a month. According to a recent study [20], a 5 mm expansion was applied to the two blood vessels: the LAD coronary artery and RCA.

The geometric similarity between SCECT and CECT contour groups was evaluated using the Dice similarity coefficient (DSC) and mean surface distance (MSD):

5 $$DSC = \frac{{2\left| {X \cap Y} \right|}}{\left| X \right| + \left| Y \right|}$$

6 $$MSD = \frac{1}{{n_{S} + n_{{S^{\prime}}} }}\left( {\mathop \sum \limits_{p = 1}^{{n_{S} }} d\left( {p,S^{\prime}} \right) + \mathop \sum \limits_{{p^{\prime} = 1}}^{{n_{{S^{\prime}}} }} d\left( {p^{\prime},S} \right)} \right)$$

Here, *X* denotes the true volume; *Y*, the predicted volume; and | |, the number of elements (voxels). *S* and *S*′ indicate the outer surfaces of the volume *X* and *Y*, respectively, *n*_*S*_ denotes the number of voxels of surface *S*, and *d*(*p*,*S*)′ is the distance between a point *p* on surface *S* and the surface *S*^′^ is given by the minimum of the Euclidean norm: $d\left( {p,S^{\prime}} \right) = \mathop {\min }\limits_{{p^{\prime} \in S^{\prime}}} p - p^{\prime}_{2}$. A higher DSC and lower MSD were associated with better values.

### Dosimetry analysis

A dosimetric evaluation was additionally performed for 20 testing datasets to determine the extent to which the difference in the contouring structure affected the final dose. Most patients included in the dataset did not receive RT; therefore, the treatment plan was transferred from those who underwent RT with similar physical geometry for this feasibility study. RayStation (RaySearch®) was used for the creation and transfer of the plan. We selected 20 plans from patients with breast cancer treated with RT. RT sites include left, right, or both breasts. All treatments were planned at 40 Gy/15 fractions. Each plan was aligned with CT-based rigid registration. For more intuitive visualization, instead of transferring the contours of SCECT and CECT to NCT, the dose distribution was transferred contrary to SCECT and CECT (Additional file 1: Figure S3). Then, we analyzed and compared the doses irradiated in the contours of the cardiac substructures of SCECT and CECT. In the overall dose-volume histogram (DVH), the D_max_, D_mean_, V5Gy, V10Gy, V20Gy, V30Gy, and V40Gy were quantitatively analyzed. All the analyses and evaluations in this study were conducted on MATLAB (The MathWorks, Inc.) based on DICOM file information.

## Results

### Image quality evaluation of SCECT

**Fig. 3:**
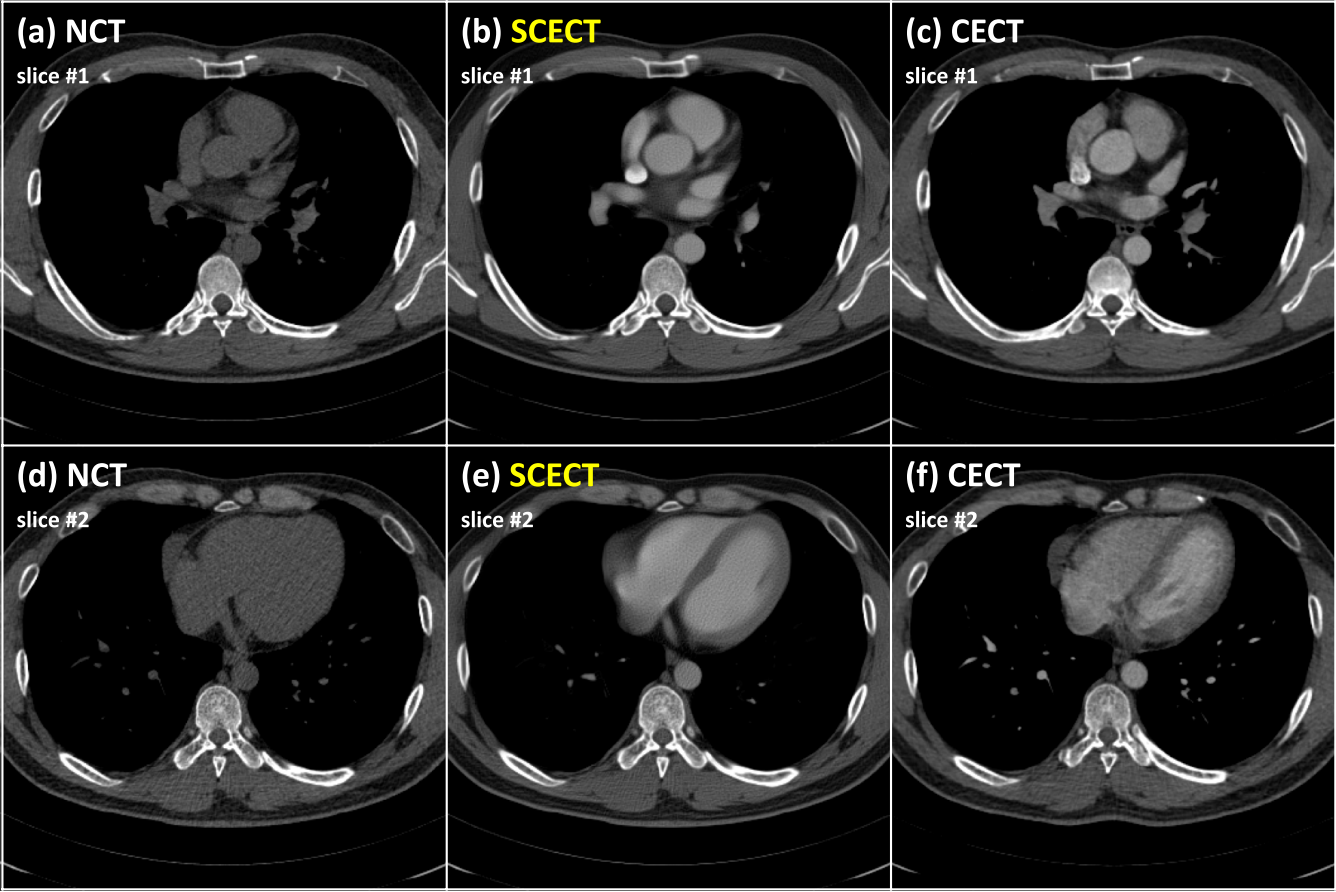
Representative images of non-contrast computed tomography (NCT) (**a**, **d**), synthetic contrast-enhanced CT (SCECT) (**b**, **e**), and contrast-enhanced CT (CECT) (**c**, **f**) of testing datasets. The upper and lower rows indicate different slices of the test datasets

Figure 3 depicts the inference results of the trained SCECT generation model. The image in the first and second rows are examples of different slices of test datasets. The SCECT generation model predicted not only the image contrast but also the virtual interventional septum structures (Fig. 2e) that were absent in NCT (Fig. 2d), similar to those of real CECT (Fig. 2f).

Additional file 1: Figure S2 depicts the MAE, SSIM, and PSNR of NCT and SCECT compared to CECT, respectively (Additional file 1). A total of 3000 slice images of 20 testing patients were used for the quantitative evaluation. While mean values (± standard deviation) of MAE, PSNR, and SSIM between SCECT and CECT were 20.66 ± 5.29, 21.57 ± 1.85, and 0.77 ± 0.06, those were 23.95 ± 6.98, 20.67 ± 2.34, and 0.76 ± 0.07 NCT and CECT, respectively. In the two-sample t-test, the results of all three indicators demonstrated statistically significant differences (*p* ≪ 0.05).

### Geometric evaluation of SCECT

**Fig. 4:**
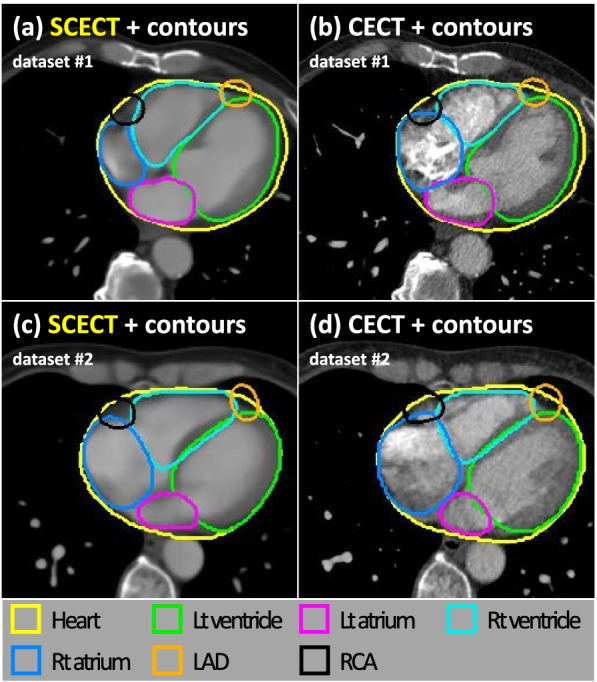
Representative results of manual contouring on synthetic contrast-enhanced CT (SCECT) (**a**, **c**) and contrast-enhanced CT (CECT) (**b**, **d**) images

Figure 4 depicts the results of manual contouring based on SCECT and CECT. The images of the first and second rows are examples of two test datasets. As a result of the quantitative analysis of contouring (Table 2), DSCs of atrial and ventricular structures showed a value higher than 0.80, and those of vascular structures showed values higher than 0.55. Overall, MSD was shorter than 3 mm except for the RCA structure.

**Table 2:** DSC and MSD statistics between manual contours of SCECT and CECT in 20 patients

Structure	Heart	Lt ventricle	Lt atrium	Rt ventricle	Rt atrium	LAD	RCA	Average
DSC	0.95 ± 0.03	0.91 ± 0.04	0.86 ± 0.08	0.85 ± 0.06	0.80 ± 0.07	0.74 ± 0.14	0.55 ± 0.20	0.81 ± 0.06
MSD (mm)	2.01 ± 0.95	1.85 ± 1.01	2.25 ± 1.03	2.41 ± 1.02	2.72 ± 0.83	2.19 ± 1.21	3.68 ± 1.60	2.44 ± 0.72

DSC, dice similarity coefficient; LAD, left anterior descending; MSD, mean surface distance; RCA, right coronary artery

### Dosimetric evaluation of SCECT

Additional file 1: Figure S3 shows overlaid images of representative test datasets with CT, contours of cardiac substructures, and the dose distribution for dosimetric evaluation (Additional file 1). Additional file 1: Figure S4 depicts an averaged DVH for all 20 test datasets (Additional file 1), and Table 3 summarizes a dosimetric analysis. The values in the table denote the absolute difference between dosimetric results of SCECT and CECT at important clinical points in the DVHs, averaged over the test datasets. Differences are displayed up to 1.56 Gy in D_max_ and 6.64% in the fractional volume in V5Gy. The MHD (D_mean_ of the heart) and V5Gy of the left ventricle (bold text in the table) demonstrated differences of 0.13 ± 0.27 Gy and 0.71 ± 1.34%, respectively. In the two-sample t-test for all distinct values in Table 3, the results did not show statistically significant differences (p ≫ 0.05).

**Table 3:** Absolute dosimetric differences between SCECT and CECT of each cardiac substructure averaged over 20 patients

Structure	D_max_	D_mean_	V5Gy	V10Gy	V20Gy	V30Gy	V40Gy
(Unit)	Gy	Gy	%	%	%	%	%
Heart	0.65 ± 1.50	**0.13** ± **0.27**	0.72 ± 1.34	0.41 ± 0.94	0.23 ± 0.66	0.16 ± 0.50	0.06 ± 0.21
Lt ventricle	0.47 ± 0.93	0.15 ± 0.33	**0.71** ± **1.34**	0.40 ± 1.16	0.21 ± 0.91	0.19 ± 0.76	0.07 ± 0.31
Lt atrium	0.25 ± 0.47	0.10 ± 0.12	0.84 ± 1.60	0.28 ± 0.85	0.00 ± 0.01	0.00 ± 0.00	0.00 ± 0.00
Rt ventricle	0.93 ± 1.60	0.21 ± 0.21	1.95 ± 2.68	0.75 ± 1.24	0.16 ± 0.47	0.08 ± 0.32	0.01 ± 0.06
Rt atrium	1.56 ± 2.90	0.28 ± 0.38	2.90 ± 4.46	0.76 ± 1.68	0.39 ± 0.97	0.14 ± 0.52	0.02 ± 0.09
LAD	0.38 ± 0.39	0.49 ± 1.10	2.81 ± 5.47	1.24 ± 3.45	1.20 ± 3.67	1.04 ± 4.02	0.03 ± 0.11
RCA	1.23 ± 1.63	0.59 ± 0.80	6.64 ± 8.57	3.61 ± 6.81	1.08 ± 3.57	1.11 ± 4.95	0.18 ± 0.82

V50Gy or higher is not displayed because most values are zero or converge to zero

CECT, contrast-enhanced computed tomography; SCECT, synthetic contrast-enhanced computed tomography; LAD, left anterior descending; RCA, right coronary artery

## Discussion

We synthesized CECT based on DL to improve the poor visualization ability of NCT. We intended to distinguish cardiac substructures through SCECT and analyze the dose-volume relationship for each substructure to enable a more meaningful retrospective cardiac toxicity study. In terms of image quality, SCECT demonstrated better similarity to CECT than NCT by quantitative metrics such as MAE, PSNR, and SSIM. Furthermore, it provided clinicians the basis for the manual contouring and modification of the cardiac substructures. A dose analysis based on the contours drawn on SCECT did not reveal a considerable difference from the actual CECT. In particular, the small error rates of 0.13 ± 0.27 Gy and 0.71 ± 1.34% were observed in MHD and V5Gy, respectively, confirming the clinical utilization of SCECT.

The final judgment on the clinical feasibility of SCECT will be made through the accuracy evaluation of the manually drawn cardiac structures. However, the SCECT generation is the ‘image translation’ task from the perspective of deep learning. The training of the model proceeds to reduce the difference between the preset SCECT and the label CECT. Metrics (MAE, PSNR, and SSIM) for image quality evaluation are used to evaluate whether the SCECT generation model has been properly learned.

We did not set up a control group such as inter-rater variation in the ‘geometric evaluation’ part of this study. Instead, in the previous study by Duane et al., [21] it was reported that inter-rater contour overlap (mean DSC) was 0.60–0.73 for left ventricular segments and 0.10–0.53 for coronary arterial segments. Interobserver contour separation was 1.5–2.2 mm for left ventricular segments and 1.3–5.1 mm for coronary artery segments in terms of directed Hausdorff average distance. This spatial variation resulted in <1 Gy dose variation for most segments but 1.2–21.8 Gy variation for segments close to a field edge. In cardiac CT images, even if we don’t consider inter-rater variation in contouring, there are intrinsic intra-fractional variations of cardiac structures, which are attributed by continuous cardiac motion and respiration motion. Cardiac-gated (ECG-gated) and respiratory-gated CT images can eliminate the major motion component, but these techniques may not be routinely used in most centers for breast RT planning or treatment. Recently, Nicolas et al. studied the heart movements in 45 patients using a cardiac-gated CT scan and suggested using 5 mm of margin surrounding the coronary artery to account for the movements in the breast RT planning [20]. We speculated that the additional 5 mm margin for RT planning might encompass the inter-rater margin.

In the absence of treatment plans for testing datasets, there can be three methods for performing dosimetric evaluation: (a) Based on the respective contours of SCECT and CECT, two optimized plans are generated and analyzed for each patient.; (b) single optimized plan is created for each patient based on NCT (or CECT) and evaluated by changing the contours only.; (c) plan transfer is performed, and dose differences are evaluated based on the respective contours. Since this study attempted to report the dose difference according to the different cardiac substructures in an actual situation in which only NCT (or CECT) exists (where only one plan exists), (a) is not appropriate. In the case of (b, c), at least one of SCECT and CECT is not optimized for the structure. Considering additional time investment (additional contouring and re-optimization), we supposed that (b) instead of (c) would not show a significant difference in analysis. In the plans of 40 Gy/15 fractions, target (hot) dose and heart are physically far enough to have an MHD of less than 5 Gy in general, so we think that the plan transfer is sufficient for this feasibility study.

Compared with the previous studies [9–13], the novel points of SCECT generation from NCT were as follows: (1) It does not cause any burden on the clinical workflow because it operates without additional medical imaging scans, such as CECT or MRI. (2) Previous AC studies without additional image acquisition have an intrinsic error because ground truth was created on NCT; in contrast, the ground truth of our model seems to be relatively reliable because it is the actual CECT. (3) It also provides clinicians with manual contouring and modification because it creates detailed cardiac substructures instead of contours themselves.

SCECT, in turn, allowed each institution to conduct a retrospective dose assessment of cardiac substructures by applying it to the NCT of abundantly existing breast RT data. The implementation of SCECT generation in actual practice would not only benefit physicians but also patients. Patients would be spared from the potential side effects of IV contrast agents. Moreover, physicians would receive additional pseudo-images similar to CECT, generated from NCT without additional image acquisition. To the best of our knowledge, this is the first study to synthesize CECT for patients undergoing breast RT using DL, besides analyzing it quantitatively and qualitatively.

This study has several limitations. First, the number of evaluation datasets is relatively small in terms of assessment in contouring and dosimetry. Since there were only 20 datasets on a patient basis, it may be challenging to extract meaningful information in evaluations performed. However, SCECT generation is ultimately an image translation task, and 3,000 slice images seem to be sufficient to evaluate the performance of the image translation model. Second, CECT acts as the ground truth of the SCECT generation model and can have different features depending on the contrast projection protocol or the elapsed time following the injection. While the overall HU value of the whole heart was high in some cases, only specific structures, such as the right ventricle, appeared exceptionally bright in others. We did not distinguish these properties in this study. Therefore, the SCECT generation model was trained to make an averaged contrast feature. If more data can be acquired in the future study, it is necessary to develop models according to characteristics such as protocol and elapsed time separately. Third, external validation using a different dataset and/or population is required to ensure the reliable performance and generalizability of the SCECT generation model.

Cardiac structures of SCECT, such as the interventional septum, are predicted artificially. Thus, they may differ from those of actual CECT. However, in terms of dosimetric analysis for cardiac toxicity studies, there was a slight difference in the important clinical points on each image group (Table 3). Considering CECT is a snapshot captured at a particular time point and averaged within the variability of patient’s breathing, motion, and heart beating, the dose differences can be interpreted to be much smaller. The assessment of contouring should be designed to meet the established endpoints. Therefore, the results of the dosimetry analysis were considered appropriate to demonstrate the feasibility of SCECT [22]. This was similar to a case in which the synthetic CT used to realize MRI-only radiotherapy demonstrated an error < 1% in dose calculation, despite the absence of 100% consistency with the actual CT [23]. We intend to continue cardiac toxicity studies according to the irradiated dose of the cardiac substructures using the present results.

## Conclusions

Our findings demonstrated the feasibility of SCECT generation from NCT and the potential for cardiac substructure delineation in target substructures, such as the ventricles, atriums, and arteries, utilizing SCECT information for breast RT. Future retrospective studies are likely to pave the way for deducing meaningful information from numerous NCTs of patients undergoing RT, which could not be utilized in the past. Moreover, this technology can not only be applied to the heart but also to various regions, such as the abdomen, for studies other than radiation toxicity.

## Supplementary Information

**Additional file 1. Fig.: S1**. Building blocks and architecture details of generator and discriminator. DB stands for Dense Block, TD stands for Transition Down, TU stands for Transition Up and $m$ corresponds to the total number of feature maps at the end of a block. **Fig. S2**. Mean absolute error (MAE), peak signal-to-noise ratio (PSNR), and structural similarity index measure (SSIM) of non-contrast computed tomography (NCT) and synthetic contrast-enhanced CT (SCECT) compared to contrast-enhanced CT (CECT), respectively. Each boxplot represents the statistics for 3,000 values of 20 testing patients in each image group. **Fig. S3**. Overlaid images of two representative patients with computed tomography, manual contours (MC), and transferred dose distribution. While the upper row (a, b) is an example of applying dose distribution of a patient with right breast cancer, the lower row (c, d) is an example of a patient with left breast cancer.; Figure S4. The averaged dose-volume histogram (DVH) over 20 patients based on synthetic contrast-enhanced computed tomography (SCECT) (dashed lines) and contrast-enhanced computed tomography (CECT) (solid lines).

## Data Availability

Data availability is limited due to institutional data protection law and confidentiality of patient data.

## Abbreviations

SCECT: Synthetic contrast-enhanced computed tomography
NCT: Non-contrast
DL: Deep learning
AC: Auto-contouring
RT: Radiation therapy
RIHD: Radiation-induced heart disease
MHD: Mean heart dose
LAD: Left anterior descending
CECT: Contrast-enhanced CT
MRI: Magnetic resonance imaging
IV: Intravenous
DCNN: Deep convolutional neural network
HU: Hounsfield units
PSNR: Peak signal-to-noise ratio
SSIM: Structural similarity index measure
RCA: Right coronary artery
DSC: Dice similarity coefficient
DVH: Dose-volume histogram
