# Supplementary material for: Synthetic contrast-enhanced computed tomography generation using a deep convolutional neural network for cardiac substructure delineation in breast cancer radiation therapy: a feasibility study
Source: Radiat Oncol. 2022 Apr 22;17:83. doi: 10.1186/s13014-022-02051-0 (PMC9034542; doi:10.1186/s13014-022-02051-0)
Supplement: Supplementary file 1 — Additional file 1. Fig.: S1. Building blocks and architecture details of generator and discriminator. DB stands for Dense Block, TD stands for Transition Down, TU stands for Transition Up and $m$m corresponds to the total number of feature maps at the end of a block. Fig. S2. Mean absolute error (MAE), peak signal-to-noise ratio (PSNR), and structural similarity index measure (SSIM) of non-contrast computed tomography (NCT) and synthetic contrast-enhanced CT (SCECT) compared to contrast-enhanced CT (CECT), respectively. Each boxplot represents the statistics for 3,000 values of 20 testing patients in each image group. Fig. S3. Overlaid images of two representative patients with computed tomography, manual contours (MC), and transferred dose distribution. While the upper row (a, b) is an example of applying dose distribution of a patient with right breast cancer, the lower row (c, d) is an example of a patient with left breast cancer.; Figure S4. The averaged dose-volume histogram (DVH) over 20 patients based on synthetic contrast-enhanced computed tomography (SCECT) (dashed lines) and contrast-enhanced computed tomography (CECT) (solid lines). [file 13014_2022_2051_MOESM1_ESM.docx]

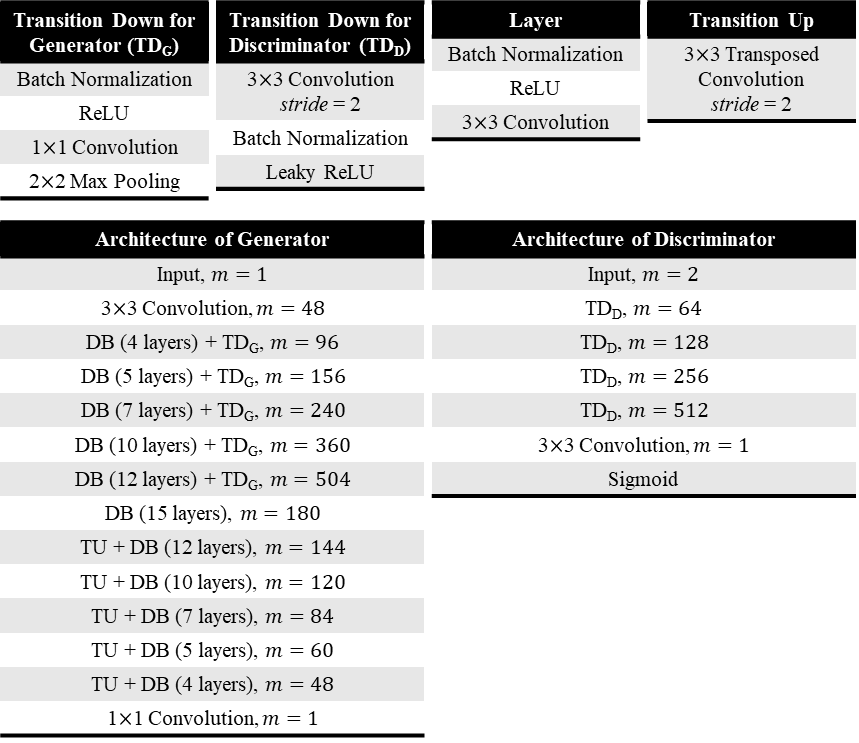


Figure S1. Building blocks and architecture details of generator and discriminator. DB stands for Dense Block, TD stands for Transition Down, TU stands for Transition Up and $m$ corresponds to the total number of feature maps at the end of a block.


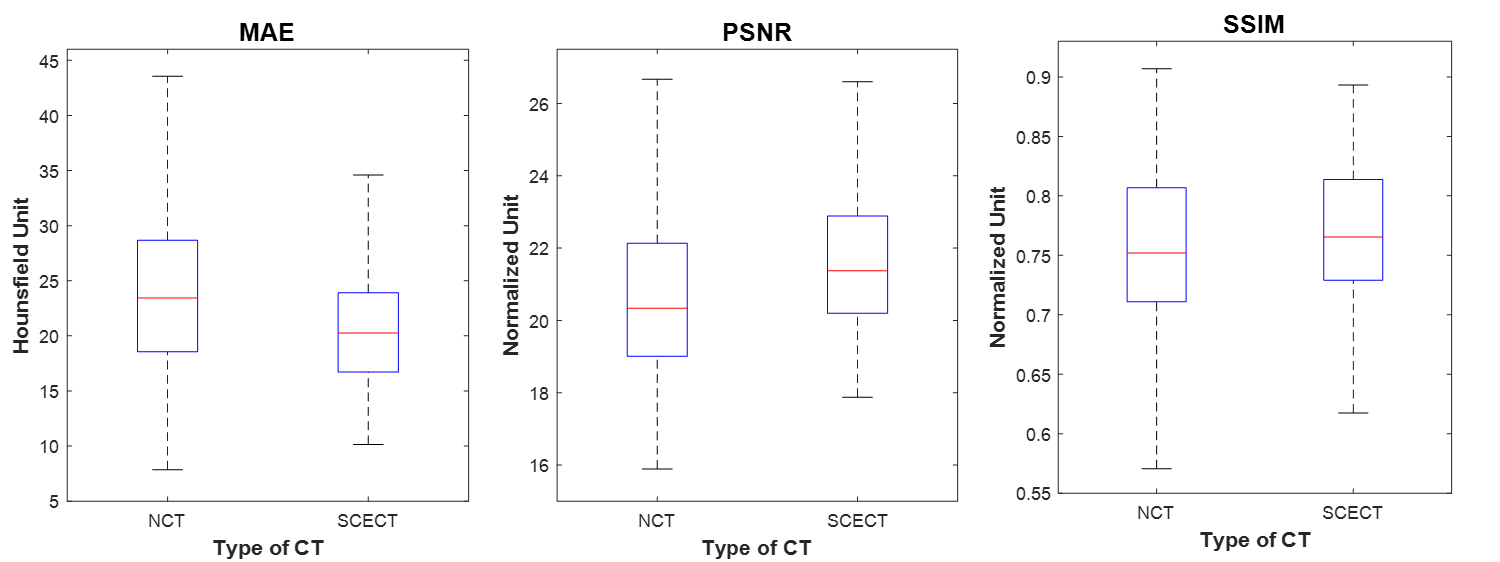


Figure S2. Mean absolute error (MAE), peak signal-to-noise ratio (PSNR), and structural similarity index measure (SSIM) of non-contrast computed tomography (NCT) and synthetic contrast-enhanced CT (SCECT) compared to contrast-enhanced CT (CECT), respectively. Each boxplot represents the statistics for 3,000 values of 20 testing patients in each image group.


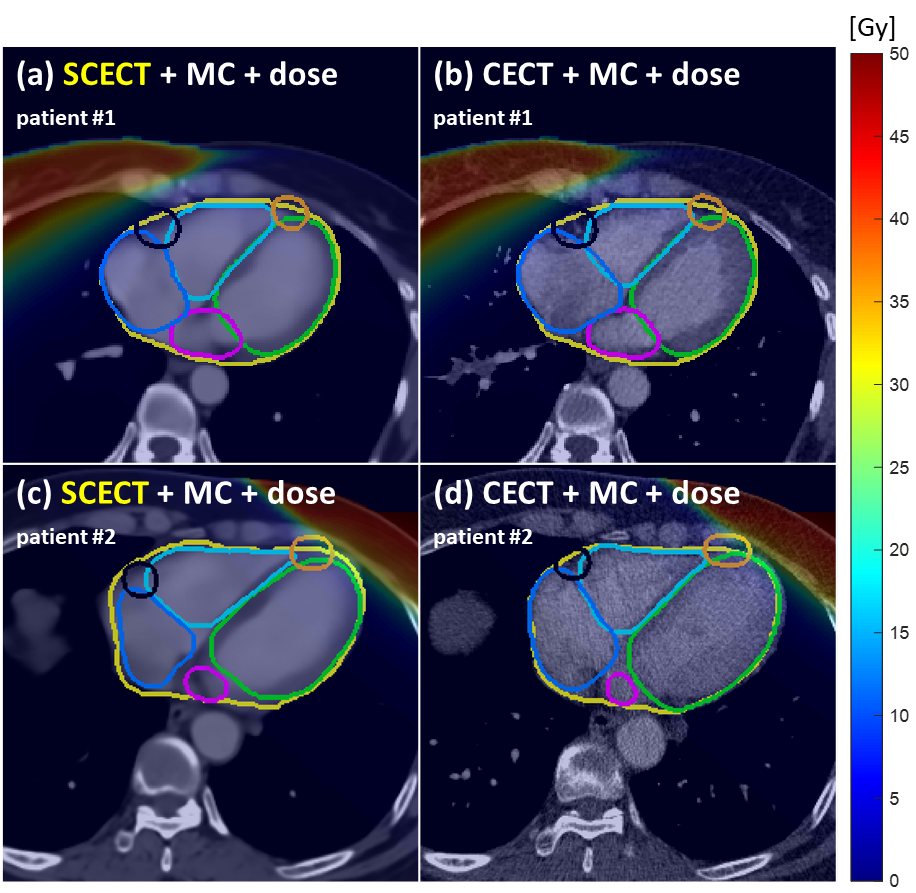


Figure S3. Overlaid images of two representative patients with computed tomography, manual contours (MC), and transferred dose distribution. While the upper row (a, b) is an example of applying dose distribution of a patient with right breast cancer, the lower row (c, d) is an example of a patient with left breast cancer.

Figure S4. The averaged dose-volume histogram (DVH) over 20 patients based on synthetic contrast-enhanced computed tomography (SCECT) (dashed lines) and contrast-enhanced computed tomography (CECT) (solid lines).
